# Interpopulation hybridization results in widespread viability selection across the genome in *Tigriopus californicus*

**DOI:** 10.1186/1471-2156-12-54

**Published:** 2011-06-03

**Authors:** Victoria L Pritchard, Leilani Dimond, J Scott Harrison, Claudia Cristina S Velázquez, Jennifer T Zieba, Ronald S Burton, Suzanne Edmands

**Affiliations:** 1 Department of Biological Sciences, University of Southern California, Los Angeles, California 90089-0371, USA; 2 Marine Biology Research Division, Scripps Institution of Oceanography, University of California at San Diego, La Jolla, California 92093-0202, USA; 3 Southwest Fisheries Science Center, 110 Shaffer Road, Santa Cruz, CA 95060-5730, USA; 4 Department of Biology, Georgia Southern University, Statesboro, Georgia 30460-8042, USA

## Abstract

**Background:**

Genetic interactions within hybrids influence their overall fitness. Understanding the details of these interactions can improve our understanding of speciation. One experimental approach is to investigate deviations from Mendelian expectations (segregation distortion) in the inheritance of mapped genetic markers. In this study, we used the copepod *Tigriopus californicus*, a species which exhibits high genetic divergence between populations and a general pattern of reduced fitness in F2 interpopulation hybrids. Previous studies have implicated both nuclear-cytoplasmic and nuclear-nuclear interactions in causing this fitness reduction. We identified and mapped population-diagnostic single nucleotide polymorphisms (SNPs) and used these to examine segregation distortion across the genome within F2 hybrids.

**Results:**

We generated a linkage map which included 45 newly elucidated SNPs and 8 population-diagnostic microsatellites used in previous studies. The map, the first available for the Copepoda, was estimated to cover 75% of the genome and included markers on all 12 *T. californicus *chromosomes. We observed little segregation distortion in newly hatched F2 hybrid larvae (fewer than 10% of markers at p < 0.05), but strikingly higher distortion in F2 hybrid adult males (45% of markers at p < 0.05). Hence, segregation distortion was primarily caused by selection against particular genetic combinations which acted between hatching and maturity. Distorted markers were not distributed randomly across the genome but clustered on particular chromosomes. In contrast to other studies in this species we found little evidence for cytonuclear coadaptation. Instead, different linkage groups exhibited markedly different patterns of distortion, which appear to have been influenced by nuclear-nuclear epistatic interactions and may also reflect genetic load carried within the parental lines.

**Conclusion:**

Adult male F2 hybrids between two populations of *T. californius *exhibit dramatic segregation distortion across the genome. Distorted loci are clustered within specific linkage groups, and the direction of distortion differs between chromosomes. This segregation distortion is due to selection acting between hatching and adulthood.

## Background

One way in which the integrity of species can be maintained is by intrinsic postzygotic barriers which reduce the fitness of hybrid offspring. Understanding how genetic interactions within interpopulation hybrids influence fitness is hence an important step towards understanding how new species arise. When gene flow between populations is restricted, they can diverge as a result of selection and drift. One outcome of this divergence may be the accumulation of 'Dobzhansky-Muller incompatibilities', mutations which only become deleterious when placed on a novel genetic background in hybrids between the populations [1,2]. As first generation (F1) hybrids contain a full haploid nuclear genome from each of the parental populations, the negative fitness consequences of Dobzhansky-Muller incompatibilities may not be expressed until the second hybrid (F2 and backcross) generations, when co-adapted blocks of the genome have been broken up by recombination. Studies have now identified regions of the genome, and in some cases specific genes, involved in such deleterious Dobzhansky-Muller interactions in hybrids [3-6]. In contrast, some authors have observed evidence for favorable interactions between divergent parental genomes in interpopulation and inter-species hybrids [7-10], suggesting that recombination of parental genomes also has the potential to increase fitness. Examination of the patterns of segregation distortion at genetic markers distributed across the genome within hybrids can both help identify candidate regions for Dobzhansky-Muller incompatibilities [11], and provide insights into other types of genetic influence on hybrid fitness.

The harpacticoid copepod *Tigriopus californicus*, an easily cultivated species that is common in supra-littoral splash pools on rocky outcrops along the west coast of North America, has become an established model for investigating the genetic basis of hybrid breakdown. There is little gene flow between populations, even those separated by relatively short distances [12,13]. Divergence in mtDNA nucleotide sequence between populations separated by less than 60 km can exceed 20%, which encompasses both synonymous and nonsynonymous sites and reflects both this low gene flow and an unusually high mtDNA substitution rate compared to nuclear genes [14,15]. The level of divergence at nuclear loci is still substantial, but is between 5.8 and 38.2-fold lower than mitochondrial divergence between the same geographic locations [14]. Despite such extreme genetic differentiation, almost all populations can easily be crossed to produce viable hybrids [16,17]. Interpopulation crosses typically exhibit a pattern of slightly increased fitness in the F1 generation, followed by lowered fitness in the F2 and later hybrid generations, as demonstrated by a variety of measurements including fecundity, development time and survivorship [18-20], response to osmotic stress [21,22], cytochrome oxidase activity [23] and mitochondrial ATP production [22]. This pattern suggests a role for Dobzhansky-Muller incompatibilities in causing the hybrid breakdown. The degree of fitness reduction in F2 hybrids shows a significant association with genetic and geographic distance between populations [16].

Much work has focused on the role of nuclear-mitochondrial incompatibilities in this hybrid breakdown. Ellison and Burton [20] found clear evidence of this intergenomic interaction. They backcrossed F3 hybrid females, which exhibited lowered fitness, to pure males from each parental population: they found that restoring a full nuclear haplotype in this way restored fitness only if the mtDNA from the matching population was also present. At least two sets of candidate loci for nuclear-mitochondrial interactions have attracted investigation. First, there has been much interest in the interaction between nuclear and mitochondrial encoded proteins involved in the oxidative phosphorylation (OXPHOS) pathway. In [24], the authors observed a general trend towards loss of fitness, associated with reduced ATP production, in hybrid lines compared to parental controls. They found that those OXPHOS enzyme complexes that contained both mitochondrial and nuclear components exhibited reduced activity in hybrid lines, whilst those containing only nuclear components did not, implicating nuclear-mitochondrial interactions in generating the observed results. Several studies have found strong evidence for nuclear-mitochondrial coadaptation affecting the activity of cytochrome c oxidase (COX), an enzyme with both nuclear and mitochondrial encoded units, and its interaction with the nuclear encoded cytochrome c [23,25,26]. Population specific interactions between COX and cytochrome c have been traced to the level of individual amino acid substitutions [27]. Second, Ellison and Burton [20] have suggested that there is also coadaptation in the mitochondrial transcription apparatus. Mitochondrial RNA polymerase (mtRPOL, nuclear-encoded) initiates mtDNA transcription by binding specific mtDNA sequence in the noncoding "control region." Using inbred hybrid lines, the authors [22] found that lines with mtRPOL and mtDNA from the same population exhibited parental OXPHOS expression patterns, whilst those with mismatching mtRPOL and mtDNA did not.

However, cytonuclear coadaptation is clearly not complete within all *T. californicus *populations [23,28] and evidence suggests that interactions within the nuclear genome also play a role in hybrid breakdown. For example, Ellison and Burton [22] found that, for hybrid lines containing mtDNA from a San Diego population (SD), parental expression was not restored with the matching mtRPOL, suggesting that at least one additional, epistatically interacting nuclear locus is present in this population but not in the experimental lines. Willett and Berkowitz [29] found dramatic segregation distortion for two nuclear encoded homologues of malic enzyme in F2 hybrids, which could be attributed to selection against particular genotypes acting between hatching and sexual maturity. Willett [30] examining segregation patterns of three nuclear-encoded OXPHOS genes, found evidence for complex nuclear epistatic interactions, but not nuclear-mitochondrial interactions, affecting survival to adulthood of F2 hybrids. Edmands and colleagues [28,31] similarly implicated epistatic interactions between different chromosomes in influencing segregation distortion in backcross and F2 hybrids.

In this study, we investigated the genetic composition of *T. californicus *interpopulation hybrids in more detail. We made crosses between two populations, SD and SC, that are 640 km apart and more than 20% divergent in mitochondrial COI and CYTB sequence [15,16]. These populations display the classic pattern of decreased survivorship of F2 interpopulation hybrids compared to parentals (Edmands and coworkers, unpublished data), and have been used in previous studies. We examined segregation pattern of eight microsatellite markers and 45 newly elucidated single nucleotide polymorphism (SNP) markers, distributed over the genome, in the F2 individuals. We compared patterns of segregation distortion between newly hatched nauplii (larvae) and adults in order to distinguish distortion caused by differential post-hatching viability from distortion caused by either meiotic drive, differential gametic fertilization success or pre-hatching mortality.

## Methods

### SNP discovery and genotyping

A cDNA library was constructed from RNA extracted from a mass copepod sample collected from the San Diego population (SD, 32º 45'N, 117º 15'W) of *T. californicus*. This sample (approximately 1 g wet weight) included adults of both sexes as well as eggs, nauplii and copepodid developmental stages. Total RNA was extracted using Tri Reagent (Sigma Chemical) using the manufacturer's protocol. Purification of mRNA from total RNA was achieved using a Qiagen mRNA isolation kit. Approximately 6 μg of mRNA was then used to create the cDNA library using the Zap Express cDNA Synthesis Kit (Stratagene), following manufacturer's protocols. Cloned cDNAs were recovered by in vivo excision of the pBluescript^® ^phagemid in *E. coli*, and 1100 positive clones (based on blue/white screening) were randomly selected for sequencing. Inserts from the positive clones were PCR amplified using M13 primers and then sequenced using T3 and T7 primers. Sequencing was carried out on an Amersham MegaBACE 500 sequencer with Amersham's ET Dye-Terminator chemistry.

From the cDNA sequences, we selected non-mitochondrial sequences over 700 bp in length and designed primers to amplify 500-700 bp portions of these sequences using Primer 3 (http://primer3.sourceforge.net/). For all primer design, we specified an optimal primer length of 20 bp and optimal melting temperature of 60ºC. We also designed primers to amplify three portions of the mitochondrial genome, using mitochondrial sequences for *T. californicus *deposited in GenBank. Primers were manufactured by Operon Biotechnologies (http://www.operon.com) and IDTDNA (http://www.idtdna.com). We tested each primer pair on two individuals from each of three populations: SD, SC (Santa Cruz, California, 36º57'N, 122º03'W), and PBJ (Punta Baja, Baja California, 36º57'N, 122º03'W, used in a separate study). DNA was extracted by placing copepods in 50 μl lysis buffer (10 mM Tris pH 8.3, 50 mM KCl, 0.5% Tween 20) with 200 μg/ml Proteinase K and incubating at 65ºC for 2 hr followed by 100ºC for 15 min. We amplified products in 25 μl reactions using the following recipe: 2.5 μl template DNA; 2.5 μl each forward and reverse primers (10 mM each); 2.5 μl premixed dNTPs (2.0 mM each); 2.0 μl MgCl_2 _(25 mM); 2.5 μl 10 X buffer, 10.4 μl DNA-free water, 0.1 μl *Taq *polymerase, and the following reaction conditions: a denaturation step of 94ºC for 5 minutes; 35 cycles of 94ºC for 30 sec, 55ºC for 30 sec, 72ºC for 30 sec (45 sec for mtDNA primers), followed by a final extension step of 72ºC for 5 minutes. PCR products were visualized on 1.8% agarose gels with ethidium bromide staining. Where a primer pair successfully produced a single amplification band in SD and at least one of the other test populations, the product was sequenced. Products were sent to the High-Throughput Genomics Unit at the University of Washington (http://www.htseq.org) for exo/sap clean-up followed by unidirectional sequencing. Two individuals per population were considered sufficient for preliminary identification of population-specific SNPs as geographically isolated *T. californicus *populations tend to be genetically homogeneous [32]. Levels of polymorphism were subsequently determined by genotyping at least 28 individuals from each of the three populations.

We used Sequencher v. 4.6 (http://www.genecodes.com) to align sequences and searched for SNPs diagnostic between both SD and SC, and SD and PBJ, with >25 bp of invariant sequence either upstream or downstream to enable the design of single base extension primers. In addition, we screened *T. californicus *mtRPOL sequence, obtained from GenBank, to identify suitable SNPs between SD and SC and SD and PBJ. We selected one suitable SNP site for each separate nuclear sequence and three from the mitochondrial sequences. SNP and flanking sequence information were provided to Jeffrey Conroy, Genomic Shared Resources, Roswell Park Cancer Institute, Buffalo, NY, who performed primer design using iPLEX Gold software (Sequenom, San Diego, CA).

All SNP genotyping was performed at Roswell Park Cancer Institute, using the iPLEX Gold Assay on a MassARRAY Compact (Sequenom). We provided unpurified, dried-down, lysis extracts (10 μl for adults, 20 μl for nauplii). Diagnostic utility of SNPs was confirmed by genotyping a larger sample of copepods from SD, SC and PBJ. Accuracy of SNP calls in heterozygotes was tested by including a blind set of known F1 SD × SC and SD × PBJ individuals. We also investigated the utility of the SNPs for *T. californicus *research in general by genotyping them for 13 additional populations, ranging from Washington to Baja California (Additional Files), the majority of which have been used in previous studies [17,32,33]

### Experimental Crosses

Reproductive biology of *T. californicus *is well established [33-35]. Males guard immature females by clasping them with their antennae until the female completes her terminal molt; the female is then inseminated and released. Unmated females are therefore easily obtained by separating clasped pairs. Females mate only once and use stored sperm to fertilize multiple clutches of eggs, with each female producing an average of ~300 progeny [35]. Inbred 'isofemale' lines are therefore established by isolating a single fertilized female and allowing offspring, including those from overlapping generations, to mate freely.

Mapping crosses were initiated using isofemale lines from populations SC and SD (one line per population) that had been inbred for 9-10 months (minimum generation time is 23 days at 20ºC [36]). All crosses were performed in 60 × 15 mm Petri dishes containing 10 ml growth medium (1 liter seawater filtered through a 37 μm filter, 0.1 g ground Tetramin fish food, 0.1 g powdered Spirulina). One virgin female from the SD line and one adult male from the SC line were placed in each Petri dish. The male was removed when the first eggsac was observed and the female moved to a new Petri dish after each eggsac hatched. The F1 offspring from each clutch were allowed to grow to maturity in the dish. When clasped pairs were observed, they were individually moved to a fresh Petri dish, where they produced the first batch of the F2 generation. Males were removed once an eggsac was observed and females again moved to a new dish after each eggsac hatched. All dishes were housed in a 20ºC incubator with a 12 h light: 12 h dark cycle. Extra Spirulina was added when supplemental food was considered necessary. As crosses were initiated using SD females, all F2 and backcross individuals contained the SD mitochondrial haplotype.

To distinguish between segregation distortion caused by meiotic drive or differential gametic fertilization success, and that caused by post-zygotic selection against particular genetic combinations, we compared results from newly-hatched nauplii to those from adult males. Newly hatched F2 nauplii for genetic analysis were obtained by removing a late-stage (orange-colored) eggsac from a female and allowing nauplii to emerge in a drop of seawater in a Petri dish. Nauplii were then killed by flooding the dish with lysis buffer and individual nauplii were transferred in 2-5 μl of liquid to a 200 μl PCR tube containing 20 μl of lysis buffer with 200 μg/ml Proteinase K. Tubes were incubated at 65ºC for 1 hr followed by 100ºC for 15 min and extracts were then stored by freezing at -70ºC. Adult males were obtained by allowing F2 families to reach maturity in the Petri dish. All males in a dish were rinsed in deionized water and frozen whole in 200 μl PCR tubes. DNA was subsequently extracted in 50 μl of lysis buffer with 200 μg/ml Proteinase K using the protocol described above. Genotyped nauplii (n = 190) were the offspring of four different F1 pairs; genotyped adult males (n = 205) were the offspring of 25 different F1 pairs.

Resources limited genotyping to only one of the two reciprocal crosses and to adults of only one gender (males). Males were chosen to eliminate the possibility of amplifying sperm or zygote alleles in fertilized females. *Tigriopus californicus *lacks heteromorphic sex chromosomes [37,38] and sex determination, although still poorly understood, may involve both additive genetic and environmental components [39,40]. Previous studies of this species have found differences between the sexes in segregation distortion [25,30,31,41], most commonly finding less distortion in males; however these differences are not systematic, varying with the populations used, direction of cross, locus and replicate. We acknowledge that focusing only on adult males limits the scope of data interpretation; as the first genome-wide assessment of larval and adult F2 genotype frequencies in this system, our study nevertheless offers important insight into the mechanism driving segregation distortion.

As female *T. californicus *do not undergo chromosomal recombination [37], we were also able to produce non-recombinant backcrosses (NR-BC) to confirm that identified linkage groups were on different chromosomes. F1 females were backcrossed to SC males. As before, NR-BC families were allowed to develop to maturity in Petri dishes and adult males (n = 39, the offspring of seven different pairs) frozen prior to genetic analysis.

### Microsatellite genotyping

In addition to the SNPs, we genotyped F2 and NR-BC individuals for nine microsatellite loci (30, 197, 228, 480, 558, 1202, 1203, 1555, 56J2 [42]) that exhibited fixed size differences between the SD and SC populations in a test sample (n = 24 each). These markers enabled SNP linkage groups to be anchored to eight of the 12 *T. californicus *chromosomes numbered by Harrison and Edmands [31]. Microsatellites were amplified individually in 12 μl reactions containing: 0.5 μl template DNA; 0.3 μl fluorescently labeled forward primer (10 mM); 1 μl reverse primer (10 mM); 1.2 μl premixed dNTPs (2.0 mM each); 1.2 μl MgCl_2 _(25 mM); 1.2 μl 10X buffer, 6.04 μl DNA-free water, 0.06 μl *Taq *polymerase, with the Thermocycler conditions as described for SNP discovery above (annealing temperature for 480 = 61ºC, for all other microsatellite loci 55ºC). PCR products were multiplexed into two pools and run on a CEQ 8000 capillary sequencer (Beckman Coulter, Fullerton, CA, USA). Allele sizes were scored manually.

### Statistical Analysis

Separate linkage maps for adults and nauplii were generated using Map Manager QTX, using the Kosambi map function with linkage criterion set at p < 0.001. We estimated corrected map length (L) and coverage (c) using the methods of [43] and [44] as follows. For each linkage group, we added two cM to the total length and then multiplied this by (m+1)/(m-1), where m is the number of markers on each group. We then estimated genomic coverage as c = 1 - e ^-2 dn/L ^where d = mean intermarker distance and n = total number of markers assigned to linkage groups. To examine segregation distortion in nauplii and adults, we compared observed single-locus genotype frequencies to those expected from Hardy-Weinberg equilibrium using the χ-squared goodness-of-fit test. Additionally, we investigated whether pairwise epistatic interactions were contributing to segregation distortion by looking at the co-occurrence of genotypic classes at different SNP loci. For each pair of physically unlinked loci we calculated expected frequencies of each genotypic combination (SDSD/SDSD; SDSD/SDSC; SDSD/SCSC; SDSC/SDSC; SDSC/SCSC; SCSC/SCSC) from the observed frequency of each genotype at each locus. We then compared observed and expected frequencies of genotypic combinations using a χ-squared goodness-of-fit test. We also used χ-squared tests to examine chromosome-level segregation distortion and pairwise linkage disequilibrium between chromosomes in the small non-recombinant backcross sample.

## Results

We generated over 20,000 bp of sequence data for each of the three *T. californicus *populations included in the SNP discovery step. Mean observed nuclear sequence divergence between SD and SC, and between SD and PBJ, was 3.8%, and 3.5% respectively. Sequence data was submitted to NCBI GenBank (Additional file 1, Table S1). We designed assays for 51 nuclear SNPs and three mitochondrial SNPs (Additional file 1, Table S1), which could be multiplexed into two pools for iPLEX Gold genotyping. Of these, 49 SNPs were identified as diagnostic between SD and SC. On the basis of results from F1 individuals, we rejected a further four SNPs from this study because SD-SC heterozygotes were undercalled, leaving 45 nuclear markers (Additional file 1, Table 1). The majority of SNPs could be scored in all but one of 14 additional *T. californicus *populations, and generally appeared fixed in each population (Additional file 1, Table S1). However, we note that most of our assays were optimized for just two alternative SNPs, meaning that the presence of additional undetected SNP alleles that did not occur in our three original populations cannot be ruled out: 2% of the total non-indel polymorphic sites observed in our sequence data exhibited three alleles. Half of all SNPs failed in PA, the only population known to exhibit reproductive isolation from others [17].

**Table 1:** Name, pre-amplification forward and reverse primers, extension primers and nucleotide calls for the 45 SNP loci used in this study

Name	Forward Primer	Reverse Primer	Extension sequence	SD	SC
**TC006**	GGGTATAGGGTTTGATCAAC	TTGAGGACCTTTTACGCAAC	AAGACCTGTGAAGAA	G	T
**TC008**	GGTTGATTGATTTTGCCTCC	TGGAGTGCAAAAGTCCATCG	GCCTCCATAGGCTCC	C	T
**TC011**	ATGAAAGACTCTTAAGTCG	GATGTCTCACAAATCTCGCC	ACTCTTAAGTCGACAGAC	T	A
**TC012**	GCAAACAGGACCGTGTTGAA	ACAAGATGGAACGGGTAGAG	CGTACACCTTGATCTTGCC	C	G
**TC016**	GTGAGTTTTGTCTCAAACGG	ATTGCCAAGCCAAGTAAGCG	TTTGTCTCAAACGGTTCACCCA	G	A
**TC017**	ATGCTTGGCAATTTGGAGTG	TTTACCTCCAGGACATTGGC	GCAATTTGGAGTGTTCTGA	C	T
**TC033**	TCCCCAAGAGCAATAATCGG	GATCGAAGTTTTGGTAGGTC	TGAGAAAATCTCCTACTTACC	C	G
**TC040**	GAGGAAGTAGAGCCAATAGC	ACCACGATGGAAGTTGCTTG	CCAATAGCAACCATCA	C	T
**TC043**	GCATTGAGGATTGTGGCTTC	TGAGGAAAAATCACGTCCGC	ATTGTGGCTTCACACCTA	C	T
**TC045**	ATTGGGATCCGACCCTGTTG	GGTTTGAACACCTGAAATGG	GGGATCTGGTTGTC	T	C
**TC046**	GGGAAAACGGGCTCAAGATG	AAAGCTGCCCTTGTAAACGC	ACAAATTGGCTTTGCTTGG	C	T
**TC051**	TCAAGCGAAACACCACCATC	TCGTACACCTGAATGGTGAC	CTTCACCACCTACTC	C	T
**TC060**	GAGGACCCATTTTTCCACAC	TCTTTTACAAGCACTCCTC	GAAGTCATCACACACG	G	A
**TC073**	ACTTGGATGTGGTGTCCTTG	CCAACACCAATGTCACCATC	GTGGTGTCCTTGGCCTCA	T	A
**TC074**	TTGCTCAAGTCAACCATGCC	GCAACCAGTTGATAGCTTCC	TCCTGTATTTTGCCT	C	G
**TC077**	GTCTCAAACGCGAATGCAAG	TTGCTTGGTTCGAGGACATC	AGAAGCCACGGCCACGTTTTC	T	C
**TC078**	GGGTACTGTGGTGTCGATTC	AAGAGGACAGCAAATGGGAC	TGTCGATTCTCCTCGG	C	G
**TC084**	GCAAATTCTGGTCCACCATC	ATGCACGTTTATGGCTACCG	TTTATCCGTACTGGTCTC	T	C
**TC085**	GAAGCCGACATCAAAATGAC	AAGGACAAATCTCCCATTGC	CATCTGAACCAGAAATCCATC	C	A
**TC099**	TAGCAATCTTCGGTCACCAC	CAGCCCATTATGATTGCTCG	GGGTTTCCACGGGATA	T	C
**TC102**	GCGTCAACGATGTTGAGAAC	TGGTGCCCATACATGAGTTC	GAACTTCATGGCCTTGAAGAT	A	T
**TC103**	GAGATCTCAGGAGAGTTGC	AATGAGGAATCTTCTCTGGC	GAGTTGCAACATCTC	T	C
**TC104**	GCTTACTTCATTCACGCACC	GAGACTTTGAGCGTCTGAAC	CCATTATTTAGTCTATCCAAGA	A	G
**TC106**	CATTTCTTCTCCCACACCAG	AATGAGAAGCAATGCTCCTG	ACGGGGATCTTGAC	C	T
**TC107**	CCTTCCCATAAAGGTCAATC	TGGAAAGAAACGGGAAGTGC	AAAGGTCAATCAATGACT	C	A
**TC111**	AAGTGTCTCTGCGAGTGCTG	TTCGAATGATGGGATCCGTG	CAACCTTCGTCGGATGTTCCCGG	C	G
**TC112**	TGGCTGCCTTTGAAGAGAAC	GAAACATCAAAGCAAGCAGG	AGAGAACATCACCTAA	C	T
**TC118**	TCAAGGACACCATCAAGAGC	TCCGGACAAGATGTTCTTGC	AGAAGGACGCCTCCTA	T	C
**TC124**	ACACCAAACCATTGAGTGGC	ATATGTAGAAGACCGAGAGC	TACCCTGTTGATGCGA	C	T
**TC125**	TTGTTGGACTTGATGCCTCG	GCTCGTATTGCTCGATCTTG	ATGTGGACAAATTGAA	G	A
**TC128**	TGTCGTATCTCGTCCTCTTC	AGAACACGAGAAGAAATTC	CTCGTCCTCTTCTTGGAATC	G	A
**TC130**	ACGTTCGTTTGTTAAGGCGG	GAACATTGCTGACAACGGAG	GTTTGTTAAGGCGGTAACTG	G	A
**TC152**	CGCCCCAGACGACGACGA	ATGCATTCGCGAAAGGCCTG	CTCCCTCAAAGCCTC	A	G
**TC155**	TTGGATCAGATCCAGCGGT	GCGAGGATTTGCAAGTACAC	AGATCCAGCGGTGCCTTAAACC	A	C
**TC156**	AATTTGATCTAGGAGCAACG	GCATTCAGTTCCAGTTTGAG	GGAGCAACGTTGCATTG	C	T
**TC157**	CAAGTTCCTGGCCAAGAAAG	TCCATGAACATGTCCATCT	GGGAACGATGATTTGGAA	G	C
**TC162**	GAAGATGCTTGAGTGGATGG	TCGGCGGAGGTGTACACTTT	AAGGCAATCTCCAGGAT	T	C
**TC167**	TGGCATCTTGATGTTTTCGC	ACTGAGGAAGGATCTTGAGG	AGGTTGGTCATTGT	T	C
**TC169**	GTAGCCCCTCAAGGTTATGC	TCACGCTTGGCAACATGGAG	AAGGGCCAATATGTGGC	T	C
**TC171**	ATGTCTCAAAACGGCCTTCC	TCGAGGAAGTTTCGTTGCAC	GCCTTCCGGATCAGCGGTA	T	C
**TC180**	AATGTCTCGCAAGAGCTGG	TGGTGGTTACCTTTCGAAAT	GAGCTGGACCCAGACAC	T	C
**TC184**	CTCCCGAATATCATGAAGGC	GGGTCCTCAACGGAAATAAC	AACCCTCAGATTTGGGAGT	G	C
**TC188**	GGTCATTCTTCGAGACCTAC	CGATACTTTTTTGAGAGTAGG	ACTCAACATGTCTGT	G	A
**TC189**	AGACGTTATGAAGCACGACC	TTTGTTATCTTCCAAGGGC	GCACGACCCAATCCGTGTC	T	A
**RPOL**	CACGTTCATTCAAAGCGGAC	AATCCAAGATTTCAACCTC	GGTTGGTAAGTCGG	G	T

Abbreviations refer to the following *T. californicus *populations: SD: Point Loma, San Diego, California (32º45'N, 117º15'W; n = 28), SC: Santa Cruz, California (36º57'N, 122º03'W; n = 28). Additional file 1, Table S1 provides information on additional SNP loci and SNP calls for additional populations.

One microsatellite (1202) was discovered to be non-diagnostic between SD and SC and was not included in the linkage map. Parents of the non-recombinant backcross individuals carried distinct alleles at this locus and therefore we were still able to use it to anchor one linkage group to a named chromosome.

**Figure 1:**
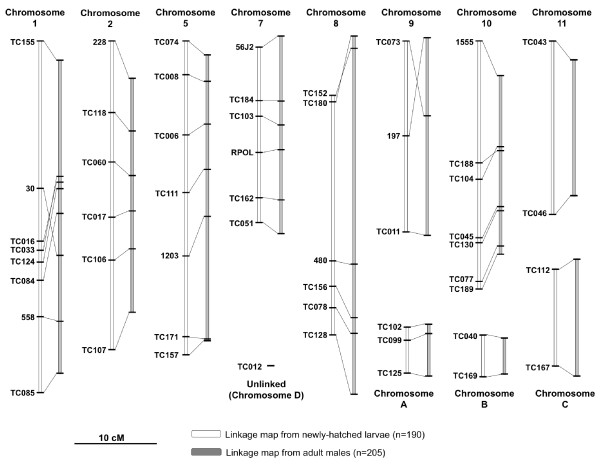
***Tigriopus californicus *linkage maps generated from an F2 mapping cross between populations SD and SC**. The maps include 45 SNPs and 8 microsatellites (1203, 56J2, 1555, 30, 228, 480, 558 and 197). Linkage groups with numerical names correspond to the numbered chromosomes identified in [31]. Maps generated from nauplii and adult males are both shown.

For both nauplii and adults, nuclear SNPs and microsatellites formed 11 linkage groups and one unlinked locus, corresponding to the 12 chromosomes of *T. californicus *(Figure 1) [37]. All linkage relationships were strongly supported, with all LOD scores greater than 11 and most much higher (Additional file 2, Table S2). However LOD scores between microsatellites markers and SNP markers were generally lower than between SNP pairs, reflecting the fact that fewer individuals were successfully genotyped for microsatellite loci (mean total n for SNP loci = 389; for microsatellite loci = 298). Results from non-recombinant backcross individuals confirmed each linkage group to be on a separate chromosome. Inclusion of the microsatellites enabled eight chromosomes named by Harrison and Edmands [31] to be identified (Figure 1). Total length of the nauplii linkage map, calculated by summing all inter-marker distances, was 266.7 cM Kosambi, with a mean between-marker distance of 6.5 cM and a maximum distance of 21.2 cM. Total adult map distance was very similar at 263.4 cM, however length differences were present between homologous linkage groups in the two maps, reflecting increased segregation distortion in the adults. Most markers were co-linear between the two maps. The greatest variation was caused by the shifting of two microsatellite markers, which may be an artifact of the smaller sample size for this marker type. Maps generated using the SNPs alone (Additional file 2, Table S2) were highly congruent, with the exception that marker TC085 was not assigned to a linkage group in the nauplii. Including the microsatellites, corrected map length for the nauplii was 484.8 cM, and estimated genomic coverage for this map was 75.2%. Corrected lengths for other maps are provided in Additional file 2, Table S2.

**Figure 2:**
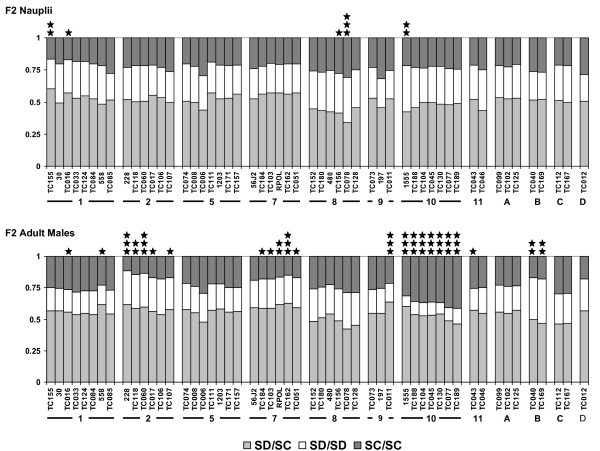
**Proportions of heterozygotes, SD homozygotes and SC homozygotes observed at each marker locus for (i) newly hatched nauplii and (ii) adult males**. The cytoplasmic background for all individuals was SD. Significance of deviations from expected 2:1:1 Mendelian segregation ratios is indicated by: ⋆⋆⋆ p < 0.00094 (Bonferroni corrected p = 0.05); ⋆⋆ p < 0.01; ⋆ p < 0.05. Markers are arranged by linkage group, as indicated under chart.

We observed little segregation distortion in F2 nauplii (Figure 2). Following Bonferroni correction for multiple tests, only one marker (TC078, on Chromosome 8) deviated significantly from Hardy-Weinberg equilibrium, with a deficiency of heterozygotes. We also observed some heterozygote deficiency at the linked marker TC156 and a deficiency of SC homozygotes at two markers on Chromosome 1. The observed deficiency of heterozygotes at microsatellite 1555 may be a technical artifact, due to a large peak size difference between the SC and SD alleles, in combination with generally lower peaks in nauplii compared to adults, causing heterozygotes to be under-called. Overall in nauplii we observed significantly more SD alleles than expected with a 1:1 ratio assuming equal genetic contributions from both parental lines (χ^2 ^= 21.3, p < 0.001).

In contrast, we observed a high level of significant segregation distortion in adult males, distributed non-randomly across the genome and biased in different directions on different chromosomes (Figure 2). Following Bonferroni correction, eleven markers deviated significantly from Hardy-Weinberg equilibrium. All markers on Chromosome 10 exhibited a very strong deficiency of SD homozygotes. There was also a deficiency of SD homozygotes, associated with an excess of SC homozygotes, at TC011 on Chromosome 9. In contrast, markers on Chromosomes 2 and 7 exhibited an excess of heterozygotes, associated with a deficiency of SC homozygotes. We also observed a deficiency of SC homozygotes and an excess of SD homozygotes at Chromosome B, although this was not significant at the Bonferroni corrected p value. In adults, we observed overall fewer SD alleles than expected with a 1:1 ratio (χ^2 ^= 21.3, p < 0.001).

**Figure 3:**
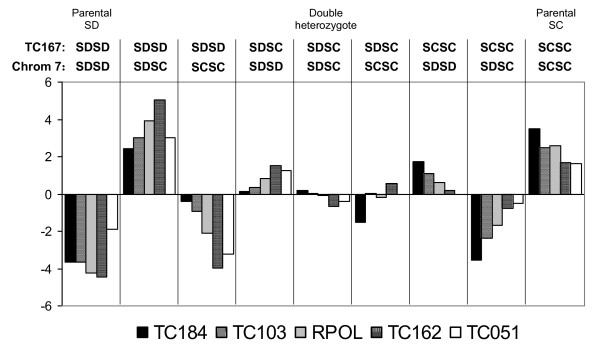
**Deviations from expected two-locus genotypic frequencies for marker TC167 and the five markers on Chromosome 7**. The y axis indicates the contribution of each possible genotypic combination, as shown above the chart, to the total χ^2 ^value. Negative value indicate that a genotypic combination was observed less frequently than expected by chance, while positive values indicate that a genotypic combination was observed more frequently. Total χ^2 ^values were as follows: TC167 - TC184, χ^2 ^= 17.1, p = 0.03; TC167 - TC103, χ^2 ^= 14.5, p = 0.07; TC167 - RPOL, χ^2 ^= 16.2, p = 0.04; TC167 - TC162, χ^2 ^= 18.3, p = 0.02; TC167 - TC051, χ^2 ^= 11.9, p = 0.16.

We observed four deviations from expected two-locus multiplicative genotypic frequencies between physically unlinked loci at p < 0.05. Although it is unclear at what level to correct for multiple testing in this case [45], as comparisons involving different physically linked markers are not independent, we note that none of these deviations were significant after a Bonferroni correction based on the number of possible comparisons between linkage groups (132). Three of these deviations involved marker TC167 on Chromosome C and markers on Chromosome 7 (TC 162, TC 184 and RPOL, Figure 3). Both individuals homozygous for SD and those homozygous for SC at Chromosome 7 markers exhibited a strong deficiency of SD homozygotes and a less marked excess of SC homozygotes at marker TC167. Those heterozygous at Chromosome 7 markers showed an excess of SD homozygotes and a deficiency of SC homozygotes at TC167. The additional significant deviation was between marker TC012 on Chromosome D and TC017 on Chromosome 2. Individuals homozygous for SC at TC012 exhibited a deficiency of SD homozygotes and an excess of heterozygotes at TC017; those homozygous for SD at TC012 showed an excess of SD homozygotes and a deficiency of heterozygotes at TC017 (χ^2 ^= 17.7, p = 0.02).

**Figure 4:**
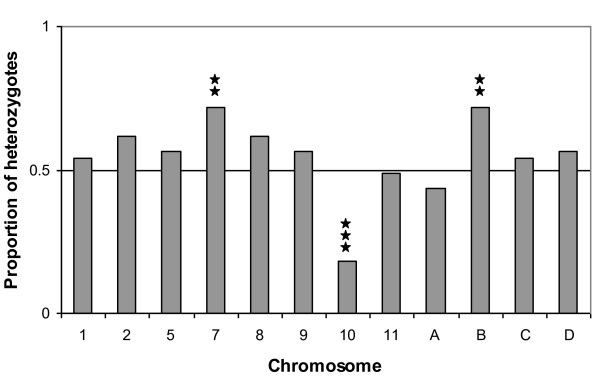
**Proportions of heterozygotes (SDSC) and homozygotes (SCSC) observed at each chromosome in adult males from the non-recombinant backcross with cytoplasmic background SD**. Significance of deviations from expected 1:1 Mendelian segregation ratios are indicated by ⋆⋆⋆ p < 0.004 (Bonferroni corrected p = 0.05); ⋆⋆ p = 0.01.

In the non-recombinant backcross, several chromosomes exhibited deviations from the expected 1:1 ratio of homozygotes (SCSC) to heterozygotes (SCSD), however only one (Chromosome 10) was significant following Bonferroni correction, probably reflecting the small sample size (Figure 4). Chromosome 10 exhibited a dramatic deficiency of heterozygotes; in contrast, heterozygote excess was observed at Chromosome 7 and Chromosome B. We found no evidence for epistatic interactions between chromosomes.

## Discussion

In this study, we mapped 45 newly developed SNP markers, and eight pre-existing microsatellite markers onto the genome of *T. californicus*. The map includes markers on all 12 *T. californicus *chromosomes and has an estimated genomic coverage of 75%. This is the first linkage map to be generated for the Copepoda, one of the most abundant animal taxa on the planet [46], and one of few currently available for crustaceans. All SNP markers are within coding loci, which will facilitate future investigations of chromosome synteny between *T. californicus *and other taxa.

We used this map to investigate patterns of segregation distortion across the genome of F2 hybrids between two *T. californicus *populations. We observed some segregation distortion in newly hatched larvae, at fewer than 10% of markers at p < 0.05, which may be caused by pre-zygotic effects or pre-hatching mortality, and was not maintained until adulthood. Segregation distortion, however, was strikingly higher in adult males, affecting 45% of marker loci at p < 0.05. This is comparable to the amount of segregation distortion that has been observed within interpopulation or inter-species crosses in other taxa (e.g. *Nasonia *spp, 29% of markers in adult males [45]; *Mimulus guttatus *48% of markers in mature plants [47]; *Arabidopsis lyrata*, 50% of markers [48]; *Daphnia magna*, 33% of markers [44]; *Daphnia pulex*, 21% of markers [49]; *Lepomis *spp. 36.8% of markers in fry [50]). As seen in other studies, distorted markers were not distributed randomly across the genome but clustered together on particular linkage groups. In *T. californicus*, this clustering may have been exacerbated by the lack of recombination in females, which increases the likelihood that markers physically linked to regions selected against in hybrids will be inherited together.

Our observation of high segregation distortion in F2 hybrid adults but not in F2 nauplii mirrors the pattern seen for several coding loci in different *T. californicus *interpopulation crosses (ME1, ME2 [29]; CYC, RISP, CYC1 [30,41]). This suggests that, in general, observed deviations from Hardy-Weinberg equilibrium in interpopulation *T. californicus *crosses are the result of selection against genotypes between hatching and adulthood, rather than being due to meiotic drive or differential gametic fertilization success. Although segregation distortion in inter-species and interpopulation hybrids is a common finding, our study is one of rather few to explicitly demonstrate a role of postzygotic selection in generating this phenomenon. Launey and Hedgecock [51] showed that segregation distortion in the oyster *Crassostrea gigas *is caused by post-hatching mortality of individuals homozygous for deleterious recessives. Martin and colleagues [52] identified hybrid genetic combinations affecting survivorship in *Iris*. Rogers and Bernatchez [53] found evidence for selection against particular genotypic combinations acting between fertilization and hatching in backcross hybrids between lake whitefish (*Coregonus clupeaformis*) ecotypes. Niehuis and colleagues [45] found evidence that cytonuclear co-adaptation caused genotypic-specific mortality between hatching and adulthood in F2 interspecific hybrids in the wasp *Nasonia*.

While we have data for only one of the two reciprocal crosses, our *T. californicus *results show very little evidence for cytonuclear coadaptation. All F2 individuals had an SD mitochondrial background: while we did observe an overall excess of SD alleles in nauplii, this had become an overall deficiency of SD alleles in adulthood. In adults we found only two markers (on Chromosome B) where the homozygote matching the SD homozygote was favored, and eight markers where the pattern of segregation distortion was opposite to that which would be expected if the nuclear and mitochondrial genomes within the two populations were co-adapted. For example, we observed dramatic segregation distortion in adult F2 males throughout Chromosome 10, with the direction of segregation distortion indicating strong selection against the nuclear genotype, SDSD, that matched the mitochondrial background. We also observed a large excess of SCSC homozygotes at Chromosome 10 in the non-recombinant backcross, suggesting that this genotype is more fit than the alternative, SDSC. Harrison and Edmands [31] correspondingly observed a deficiency of SDSD Chromosome 10 homozygotes in males (but not in females) in backcrosses between SD and another population, RP (Royal Palms). Taken together, these results suggest that part or all of Chromosome 10 derived from the SD population has a deleterious effect on viability in males of this interpopulation cross, that appears to act in an incompletely dominant manner. There are several reasons why such an apparently deleterious portion of the genome may be maintained in the SD population. First, this deleterious effect may be only expressed in a hybrid nuclear genetic background. Our study did not detect epistatic interactions involving Chromosome 10, although this may be due to limited power. Second, as we only examined males, it is possible that aspects of SD Chromosome 10 may be advantageous in females. Alternatively, genes on SC Chromosome 10 may cause masculinization; we note, however, that while F2 offspring in this study did not deviate from a 1:1 sex ratio, the concurrently generated nonrecombinant backcross was significantly female biased despite all individuals containing at least one copy of the SC chromosome (V.L. Pritchard and coworkers, unpublished data). Third, the deleterious aspect of Chromosome 10 may not be expressed in the natural environment. It may be masked in the wild SD population by the presence of a more dominant allele that was lost both from our isofemale lines and the SD parental line used in [31]. Even if this is not the case, previous studies with *T. californicus *have shown varying experimental conditions to alter the viability of different hybrid genotypes [26,27,54]. Additionally, the outcome of replicated experimental hybridizations may vary even under apparently identical conditions [41], suggesting that even apparently minor environmental changes can have a large influence on the fitness of different genotypes. Finally, T. *californicus *populations in the wild experience repeated population bottlenecks, which are expected to affect the outcome of selection. Hence even if the deleterious aspect of Chromosome 10 is expressed in the natural environment it may persist in the wild SD population due to drift. Indeed, there is evidence that many *T. californicus *populations carry such a genetic load [28]. For the SD population in particular, previous studies have indicated a selective disadvantage to SD homozygotes for the coding loci ME2 and CYC, even on the SD mitochondrial background [29,41]. In contrast, SD homozygotes were favored on a mismatching mitochondrial background, for the coding loci ME1 and RISP [29,41]. We note, however, that these results vary by sex and study, and none of these four coding loci are on linkage groups exhibiting significant segregation distortion in the current cross (ME1, Chromosome C; ME2, Chromosome A; CYC, Chromosome D; RISP, Chromosome 8, Rose and Edmands, unpublished data). Studies of taxa other than *T. californicus *have also shown that homozygotes mismatching the cytoplasmic background can be favored in hybrids. Fishman and colleagues [55], for example, examining segregation distortion in *Mimulus *F2 hybrids, found a strong excess of *M. guttatus *homozygotes on a *M. nasutus *cytoplasmic background. Similarly Martin and colleagues [52], in a backcross study using *Iris brevicaulis *and *I. fulva*, found that, at three QTLs, presence of *I. fulva *homozygotes decreased long-term survivorship despite a matching cytoplasmic background.

In contrast to the pattern observed for Chromosome 10, we observe heterozygote excess, with no apparent selection against SDSD homozygotes, throughout most of Chromosome 2 and Chromosome 7. We also observe an excess of heterozygotes for Chromosome 7 in the nonrecombinant backcross. This chromosome contains the locus coding for mtRPOL, which has been the focus of recent studies of cytonuclear coadaptation in *T. californicus*. In comparison to our results, Ellison and Burton [22], looking at allelic frequencies in F4 hybrid adults, found evidence for selection against the SD mtRPOL genotype in crosses with both SC and another population, AB. They also observed that, unlike for other crosses, recombinant inbred lines with matching SD mtRPOL and SD mtDNA did not demonstrate the same OXPHOS transcriptional profile under conditions of hypo-osmotic stress as SD parentals, suggesting that an epistatically interacting nuclear locus is involved in mitochondrial transcription in SD. They suggested the transcription factor TFAM as a possible candidate. In this context, it is interesting that our results are suggestive of an epistatic interaction between marker TC167 and Chromosome 7; recent work (Rose and Edmands, unpublished) has revealed marker TC167 to be closely linked to TFAM. Nevertheless, the genotypic association patterns between mtRPOL and TC167 are not what would be expected if there is simple co-adaptation between SD mtRPOL and SD TFAM; individuals homozygous for SD mtRPOL exhibit a deficiency of SD homozygotes, and a slight excess of SC homozygotes, at TC167.

Higher divergence between parental lines is expected to result in increased frequencies of distorted loci [56], particularly due to heterozygote deficits [49]. For example, parental divergence is cited as the reason why crosses between mildly divergent *D. pulex *populations result in 21% of markers showing segregation distortion, largely due to homozygote deficits [49], while crosses between highly divergent *Daphnia magna *populations result in 33% of markers showing transmission ratio distortion, largely due to heterozygote deficits [44]. This pattern is consistent with the prediction that overdominance between alleles of closely related taxa may yield to underdominance between alleles in more distantly-related taxa [57]. Alternatively, divergence may increase the ratio of epistatic interactions involving heterozygous loci. In the current study hybridization between highly differentiated populations (over 20% mitochondrial divergence [15,16]) led to a high frequency of marker distortion in adults (45%), but no significant heterozygote deficits, indicating relatively slow accumulation of underdominance and/or epistasis involving heterozygotes.

As has been seen in other *Tigriopus *studies [30], and in other taxa [58] it is clear that nuclear loci can interact in a complex way to influence fitness; unfortunately we lack the power to investigate such interactions in more depth in the current study. Additionally, we did not consider epigenetic effects, which have previously been suggested to alter gene transcription in interpopulation hybrids of *T. californicus *[59]. Overall, our results suggest many intriguing avenues for further investigation into the genetic basis of reduced fitness in interpopulation hybrids of *T. californicus*. These studies will be greatly facilitated by the recent transcriptome assembly for both the SD and SC *T. californicus *populations [60], and by continuing advances in crustacean genomics [61].

## Conclusion

We developed 45 population-diagnostic SNP markers for *Tigriopus californicus*, with which we generated the first linkage map available for the Copepoda. We used this to examine segregation distortion in F2 interpopulation hybrids, which are known to have reduced fitness compared to parental populations. We found dramatic segregation distortion in adult males, but not in newly hatched larvae, indicating that this distortion arises as a result of selection against particular genotypic combinations between hatching and adulthood. Distorted markers were not distributed randomly across the genome but clustered within particular linkage groups. In contrast to other studies, we found little evidence for cytonuclear co-adaptation in this interpopulation cross. Instead, different linkage groups exhibited markedly different patterns of distortion, that appear to have been influenced by nuclear-nuclear epistatic interactions and may also reflect genetic load carried within the parental lines.

## List of abbreviations

ATP: adenosine triphosphate; cDNA: complementary deoxyribonucleic acid; CYC: cytochrome c; dNTP: deoxyribonucleotide triphosphate; LOD: logarithm of the odds; ME: malic enzyme; mRNA: messenger ribonucleic acid; PCR: polymerase chain reaction; QTL: quantitative trait locus; RISP: rieske iron-sulfur protein.

## Authors' contributions

VLP performed SNP development, *Tigriopus *crosses, microsatellite genotyping, statistical analyses, and wrote the manuscript. SE designed the study and assisted with crosses. JSH and RSB generated EST data. JTZ, LD and CCSV assisted with SNP development and microsatellite genotyping. SE, JSH and RSB contributed to the manuscript, which all authors approved prior to submission.

## Supplementary Material

Additional file 1 **Table S1: Name, accession numbers, primers, SNP calls and Blast matches for 51 nuclear and 3 mitochondrial loci**. Abbreviations refer to the following *T. californicus *populations: SD: Point Loma, San Diego, California (32º45'N, 117º15'W, n = 28); SC: Santa Cruz, California (36º57'N, 122º03'W, n = 28); PBJ: Punta Baja, Baja California (29º58'N, 115º48'W, n = 31); SUN: Sunset Beach, Washington (48º30'N, 123º00'W, n = 20); FHL: Friday Harbor Laboratories, Washington (48º33'N, 125º08'W, n = 15); LC: Leo Carillo Beach, California (34º03'N, 118º56 W, n = 20); PD: Point Dume, California (34º00'N, 118º48'W, n = 18); CAT: Catalina Island, California (33º27'N, 118 29'W, n = 19); SCI: Santa Cruz Island, California (34º03'N, 119º34'W, n = 17); RP: Royal Palms, Palos Verdes, California (33º42'N, 118º19'W, n = 13); AB: Abalone Cove, Palos Verdes, California (34º44'N, 118º19'W, n = 20); LB: Laguna Beach, California (33º33'N, 117º47'W, n = 19); OSP: Osprey Point, San Diego, California (32º44'N, 117 º15'W, n = 16); PBN: Punta Banda, Baja California (31º43'N, 116º43'W, n = 19); PMO: Punta Morro, Baja California (31º52'N, 116º40'W, n = 20); PA: Playa Altamira, Baja California (28º33'N, 114º05'W, n = 20). An 'n' indicates successful SNP calls in less than 80% of the individuals screened. Loci marked with * exhibit a deficiency of identified heterozygotes in known SD × SC F1 individuals (under-calling). 'Genbank # (primer design)' refers to the accessions from which sequencing primers were designed. 'BLAST match' shows results of a BlastX search of the NCBI protein database using these sequences; only matches with an E-value < 1 × E^-5 ^are shown. 'Genbank # (sequence)' references the sequences from which the SNPs were identified. Click here for file

Additional file 2 **Table S2: Number of inter-locus comparisions (n), map distance in cM Kosambi (Distance), standard error of map distance (SE), and LOD scores for all markers on the twelve chromosomes (Chrom)**. Data are shown for nauplii and adults, both including and omitting microsatellite markers. Total map lengths and map lengths corrected following [43,44] are also provided. Click here for file
