# Supplementary material for: Interpopulation hybridization results in widespread viability selection across the genome in Tigriopus californicus
Source: BMC Genet. 2011 Jun 3;12:54. doi: 10.1186/1471-2156-12-54 (PMC3138442; doi:10.1186/1471-2156-12-54)
Supplement: Additional file 2 — Table S2: Number of inter-locus comparisions (n), map distance in cM Kosambi (Distance), standard error of map distance (SE), and LOD scores for all markers on the twelve chromosomes (Chrom). Data are shown for nauplii and adults, both including and omitting microsatellite markers. Total map lengths and map lengths corrected following [43,44] are also provided. [file 1471-2156-12-54-S2.DOC]

|  | Nauplii all markers | |  |  |  |  |  | Nauplii SNPs only | |  |  |  |  |
| --- | --- | --- | --- | --- | --- | --- | --- | --- | --- | --- | --- | --- | --- |
| Chrom | Locus 1 | Locus 2 | n | Distance | SE | LOD |  | Locus 1 | Locus 2 | n | Distance | SE | LOD |
|  |  |  |  |  |  |  |  |  |  |  |  |  |  |
| 1 | TC155 | 30 | 158 | 17.9 | 2.7 | 18.0 |  | TC155 | TC016 | 184 | 14.3 | 2.1 | 25.3 |
| 1 | 30 | TC016 | 159 | 6.6 | 1.5 | 40.9 |  | TC016 | TC033 | 180 | 1.1 | 0.6 | 69.6 |
| 1 | TC016 | TC033 | 180 | 1.1 | 0.6 | 69.6 |  | TC033 | TC124 | 182 | 1.4 | 0.6 | 69.5 |
| 1 | TC033 | TC124 | 182 | 1.4 | 0.6 | 69.5 |  | TC124 | TC084 | 187 | 2.2 | 0.8 | 66.9 |
| 1 | TC124 | TC084 | 187 | 2.2 | 0.8 | 66.9 |  |  |  |  |  |  |  |
| 1 | TC084 | 558 | 152 | 4.4 | 1.2 | 47.5 |  |  | TC085 |  | Unlinked |  |  |
| 1 | 558 | TC085 | 138 | 9.2 | 1.9 | 30.5 |  |  |  |  |  |  |  |
|  |  |  |  |  |  |  |  |  |  |  |  |  |  |
| 2 | 228 | TC118 | 147 | 8.7 | 1.8 | 32.2 |  | TC118 | TC060 | 188 | 6.1 | 1.3 | 51.7 |
| 2 | TC118 | TC060 | 188 | 6.1 | 1.3 | 51.7 |  | TC060 | TC017 | 187 | 6.7 | 1.4 | 47.3 |
| 2 | TC060 | TC017 | 187 | 6.7 | 1.4 | 47.3 |  | TC017 | TC106 | 188 | 5.2 | 1.2 | 52.6 |
| 2 | TC017 | TC106 | 188 | 5.2 | 1.2 | 52.6 |  | TC106 | TC107 | 185 | 11.1 | 1.8 | 35.5 |
| 2 | TC106 | TC107 | 185 | 11.1 | 1.8 | 35.5 |  |  |  |  |  |  |  |
|  |  |  |  |  |  |  |  |  |  |  |  |  |  |
| 5 | TC074 | TC008 | 189 | 4.1 | 1.0 | 60.0 |  | TC074 | TC008 | 189 | 4.1 | 1.0 | 66.0 |
| 5 | TC008 | TC006 | 169 | 7.4 | 1.5 | 44.4 |  | TC008 | TC006 | 169 | 7.4 | 1.5 | 26.0 |
| 5 | TC006 | TC111 | 164 | 7.0 | 1.5 | 43.1 |  | TC006 | TC111 | 164 | 7.0 | 1.5 | 43.1 |
| 5 | TC111 | 1203 | 165 | 7.6 | 1.6 | 38.7 |  | TC111 | TC171 | 184 | 14.6 | 2.2 | 26.0 |
| 5 | 1203 | TC171 | 168 | 9.9 | 1.8 | 34.6 |  | TC171 | TC157 | 187 | 2.2 | 0.8 | 66.0 |
| 5 | TC171 | TC157 | 187 | 2.2 | 0.8 | 66.0 |  |  |  |  |  |  |  |
|  |  |  |  |  |  |  |  |  |  |  |  |  |  |
| 7 | 56J2 | TC184 | 144 | 6.5 | 1.5 | 36.8 |  | TC184 | TC103 | 188 | 1.9 | 0.7 | 67.1 |
| 7 | TC184 | TC103 | 188 | 1.9 | 0.7 | 67.1 |  | TC103 | RPOL | 188 | 4.4 | 1.1 | 54.7 |
| 7 | TC103 | RPOL | 188 | 4.4 | 1.1 | 54.7 |  | RPOL | TC162 | 187 | 5.5 | 1.2 | 50.2 |
| 7 | RPOL | TC162 | 187 | 5.5 | 1.2 | 50.2 |  | TC162 | TC051 | 187 | 3.0 | 0.9 | 61.0 |
| 7 | TC162 | TC051 | 187 | 3.0 | 0.9 | 61.0 |  |  |  |  |  |  |  |
|  |  |  |  |  |  |  |  |  |  |  |  |  |  |
| 8 | TC152 | TC180 | 185 | 0.8 | 0.5 | 79.7 |  | TC152 | TC180 | 185 | 0.8 | 0.5 | 79.7 |
| 8 | TC180 | 480 | 169 | 19.3 | 2.7 | 22.4 |  | TC180 | TC156 | 186 | 19.0 | 2.6 | 25.2 |
| 8 | 480 | TC156 | 166 | 3.1 | 1.0 | 60.3 |  | TC156 | TC078 | 153 | 2.6 | 0.9 | 61.9 |
| 8 | TC156 | TC078 | 153 | 2.6 | 0.9 | 61.9 |  | TC078 | TC128 | 155 | 3.3 | 1.0 | 59.1 |
| 8 | TC078 | TC128 | 155 | 3.3 | 1.0 | 59.1 |  |  |  |  |  |  |  |
|  |  |  |  |  |  |  |  |  |  |  |  |  |  |
| 9 | TC073 | 197 | 160 | 11.5 | 2.0 | 30.4 |  | TC073 | TC011 | 190 | 15.0 | 2.2 | 27.6 |
| 9 | 197 | TC011 | 160 | 11.8 | 2.0 | 30.1 |  |  |  |  |  |  |  |
|  |  |  |  |  |  |  |  |  |  |  |  |  |  |
| 10 | 1555 | TC188 | 144 | 14.8 | 2.5 | 24.3 |  | TC188 | TC104 | 180 | 2.0 | 0.7 | 68.4 |
| 10 | TC188 | TC104 | 180 | 2.0 | 0.7 | 68.4 |  | TC104 | TC045 | 182 | 7.2 | 1.4 | 46.4 |
| 10 | TC104 | TC045 | 182 | 7.2 | 1.4 | 46.4 |  | TC045 | TC130 | 187 | 0.5 | 0.4 | 79.8 |
| 10 | TC045 | TC130 | 187 | 0.5 | 0.4 | 79.8 |  | TC130 | TC077 | 186 | 4.7 | 1.1 | 57.7 |
| 10 | TC130 | TC077 | 186 | 4.7 | 1.1 | 57.7 |  | TC077 | TC189 | 185 | 1.1 | 0.5 | 75.6 |
| 10 | TC077 | TC189 | 185 | 1.1 | 0.5 | 75.6 |  |  |  |  |  |  |  |
|  |  |  |  |  |  |  |  |  |  |  |  |  |  |
| 11 | TC043 | TC046 | 183 | 21.2 | 2.9 | 19.5 |  | TC043 | TC046 | 183 | 21.2 | 2.9 | 19.5 |
|  |  |  |  |  |  |  |  |  |  |  |  |  |  |
| A | TC102 | TC99 | 190 | 1.6 | 0.6 | 71.5 |  | TC102 | TC99 | 190 | 1.6 | 0.6 | 71.5 |
| A | TC99 | TC125 | 190 | 4.0 | 1.0 | 58.7 |  | TC99 | TC125 | 190 | 4.0 | 1.0 | 58.7 |
|  |  |  |  |  |  |  |  |  |  |  |  |  |  |
| B | TC040 | TC169 | 188 | 5.2 | 1.2 | 54.1 |  | TC040 | TC169 | 188 | 5.2 | 1.2 | 54.1 |
|  |  |  |  |  |  |  |  |  |  |  |  |  |  |
| C | TC112 | TC167 | 186 | 11.9 | 1.9 | 34.2 |  | TC112 | TC167 | 186 | 11.9 | 1.9 | 34.2 |
|  |  |  |  |  |  |  |  |  |  |  |  |  |  |
| D | TC012 |  |  | unlinked |  |  |  | TC012 |  |  | unlinked |  |  |
|  |  |  |  |  |  |  |  |  |  |  |  |  |  |
|  | Total map length | | | 266.7 |  |  |  | Total map length | | | 198.3 |  |  |
|  | Corrected map length | | | 484.8 |  |  |  | Corrected map length | | | 420.7 |  |  |
|  | | |  |  |  |  |  |  | | |  |  |  |

|  | Adults all markers | |  |  |  |  |  | Adults SNPs only | |  |  |  |  |
| --- | --- | --- | --- | --- | --- | --- | --- | --- | --- | --- | --- | --- | --- |
| Chrom | Locus 1 | Locus 2 | n | Distance | SE | LOD |  | Locus 1 | Locus 2 | n | Distance | SE | LOD |
|  |  |  |  |  |  |  |  |  |  |  |  |  |  |
| 1 | TC155 | TC033 | 201 | 14.1 | 2.0 | 29.6 |  | TC155 | TC033 | 201 | 14.1 | 2.0 | 29.6 |
| 1 | TC033 | TC016 | 201 | 0.7 | 0.4 | 80.5 |  | TC033 | TC016 | 201 | 0.7 | 0.4 | 80.5 |
| 1 | TC016 | TC124 | 204 | 0.7 | 0.4 | 81.7 |  | TC016 | TC124 | 204 | 0.7 | 0.4 | 81.7 |
| 1 | TC124 | TC084 | 204 | 3.0 | 0.9 | 67.7 |  | TC124 | TC084 | 204 | 3.0 | 0.9 | 67.7 |
| 1 | TC084 | 30 | 163 | 5.1 | 1.3 | 45.2 |  | TC084 | TC085 | 198 | 10.9 | 1.7 | 37.2 |
| 1 | 30 | 558 | 113 | 8.0 | 1.9 | 23.7 |  |  |  |  |  |  |  |
| 1 | 558 | TC085 | 124 | 14.0 | 2.6 | 16.6 |  |  |  |  |  |  |  |
|  |  |  |  |  |  |  |  |  |  |  |  |  |  |
| 2 | 228 | TC118 | 147 | 6.4 | 1.5 | 34.6 |  | TC118 | TC060 | 203 | 5.4 | 1.2 | 53.4 |
| 2 | TC118 | TC060 | 203 | 5.4 | 1.2 | 53.4 |  | TC060 | TC017 | 202 | 4.3 | 1.0 | 58.6 |
| 2 | TC060 | TC017 | 202 | 4.3 | 1.0 | 58.6 |  | TC017 | TC106 | 202 | 4.6 | 1.1 | 59.0 |
| 2 | TC017 | TC106 | 202 | 4.6 | 1.1 | 59.0 |  | TC106 | TC107 | 205 | 7.7 | 1.4 | 47.3 |
| 2 | TC106 | TC107 | 205 | 7.7 | 1.4 | 47.3 |  |  |  |  |  |  |  |
|  |  |  |  |  |  |  |  |  |  |  |  |  |  |
| 5 | TC074 | TC008 | 205 | 3.2 | 0.9 | 65.6 |  | TC074 | TC008 | 205 | 3.2 | 0.0 | 65.6 |
| 5 | TC008 | TC006 | 188 | 5.2 | 1.2 | 55.0 |  | TC008 | TC006 | 188 | 5.2 | 1.2 | 55.0 |
| 5 | TC006 | TC111 | 187 | 5.5 | 1.2 | 53.2 |  | TC006 | TC111 | 187 | 5.5 | 1.2 | 53.2 |
| 5 | TC111 | 1203 | 165 | 5.7 | 1.3 | 42.0 |  | TC111 | TC171 | 293 | 10.1 | 1.6 | 39.3 |
| 5 | 1203 | TC171 | 167 | 14.9 | 2.3 | 21.7 |  | TC171 | TC157 | 201 | 0.2 | 0.2 | 84.0 |
| 5 | TC171 | TC157 | 201 | 0.2 | 0.2 | 84.0 |  |  |  |  |  |  |  |
|  |  |  |  |  |  |  |  |  |  |  |  |  |  |
| 7 | 56J2 | TC184 | 134 | 7.9 | 1.8 | 29.2 |  | TC184 | TC103 | 205 | 4.1 | 1.0 | 65.6 |
| 7 | TC184 | TC103 | 205 | 3.0 | 0.9 | 65.6 |  | TC103 | RPOL | 205 | 6.1 | 1.2 | 64.4 |
| 7 | TC103 | RPOL | 205 | 3.0 | 0.9 | 64.4 |  | RPOL | TC162 | 205 | 3.0 | 0.9 | 49.3 |
| 7 | RPOL | TC162 | 205 | 6.1 | 1.2 | 49.3 |  | TC162 | TC051 | 203 | 3.0 | 0.9 | 58.4 |
| 7 | TC162 | TC051 | 203 | 4.1 | 1.0 | 58.4 |  |  |  |  |  |  |  |
|  |  |  |  |  |  |  |  |  |  |  |  |  |  |
| 8 | TC180 | TC152 | 199 | 1.5 | 0.6 | 77.7 |  | TC152 | TC180 | 199 | 1.5 | 0.6 | 77.7 |
| 8 | TC152 | 480 | 158 | 26.2 | 3.7 | 11.2 |  | TC180 | TC156 | 204 | 26.1 | 3.3 | 15.1 |
| 8 | 480 | TC156 | 160 | 6.5 | 1.4 | 42.0 |  | TC156 | TC078 | 177 | 4.2 | 1.2 | 56.2 |
| 8 | TC156 | TC078 | 177 | 5.2 | 1.2 | 56.2 |  | TC078 | TC128 | 173 | 4.1 | 1.1 | 60.4 |
| 8 | TC078 | TC128 | 173 | 4.1 | 1.1 | 60.4 |  |  |  |  |  |  |  |
|  |  |  |  |  |  |  |  |  |  |  |  |  |  |
| 9 | 197 | TC073 | 130 | 8.6 | 1.9 | 27.8 |  | TC073 | TC011 | 201 | 14.9 | 2.1 | 26.3 |
| 9 | TC073 | TC011 | 201 | 14.9 | 2.1 | 26.3 |  |  |  |  |  |  |  |
|  |  |  |  |  |  |  |  |  |  |  |  |  |  |
| 10 | 1555 | TC104 | 141 | 8.7 | 1.8 | 29.8 |  | TC104 | TC188 | 203 | 0.5 | 0.3 | 84.5 |
| 10 | TC104 | TC188 | 203 | 0.5 | 0.3 | 84.5 |  | TC188 | TC045 | 200 | 6.8 | 1.3 | 50.2 |
| 10 | TC188 | TC045 | 200 | 6.8 | 1.3 | 50.2 |  | TC045 | TC130 | 201 | 0.5 | 0.4 | 83.3 |
| 10 | TC045 | TC130 | 201 | 0.5 | 0.4 | 83.3 |  | TC130 | TC077 | 204 | 4.3 | 1.0 | 63.3 |
| 10 | TC130 | TC077 | 204 | 4.3 | 1.0 | 63.3 |  | TC077 | TC189 | 200 | 1.0 | 0.5 | 83.0 |
| 10 | TC077 | TC189 | 200 | 1.0 | 0.5 | 83.0 |  |  |  |  |  |  |  |
|  |  |  |  |  |  |  |  |  |  |  |  |  |  |
| 11 | TC043 | TC046 | 202 | 16.6 | 2.3 | 24.8 |  | TC043 | TC046 | 202 | 16.6 | 2.3 | 24.8 |
|  |  |  |  |  |  |  |  |  |  |  |  |  |  |
| A | TC102 | TC99 | 203 | 1.0 | 0.5 | 79.4 |  | TC102 | TC099 | 203 | 1.0 | 0.5 | 79.4 |
| A | TC099 | TC125 | 201 | 5.4 | 1.2 | 54.0 |  | TC099 | TC125 | 201 | 5.4 | 1.2 | 54.0 |
|  |  |  |  |  |  |  |  |  |  |  |  |  |  |
| B | TC040 | TC169 | 204 | 4.5 | 1.1 | 64.1 |  | TC040 | TC169 | 204 | 4.5 | 1.1 | 64.1 |
|  |  |  |  |  |  |  |  |  |  |  |  |  |  |
| C | TC112 | TC167 | 199 | 14.3 | 2.0 | 33.6 |  | TC112 | TC167 | 199 | 14.3 | 2.0 | 33.6 |
|  |  |  |  |  |  |  |  |  |  |  |  |  |  |
| D | TC012 |  |  | unlinked |  |  |  | TC012 |  |  | unlinked |  |  |
|  |  |  |  |  |  |  |  |  |  |  |  |  |  |
|  | Total map length | | | 263.4 |  |  |  | Total map length | | | 197.5 |  |  |
|  | Corrected map length | | | 476.4 |  |  |  | Corrected map length | | | 413.6 |  |  |
|  |  |  |  |  |  |  |  |  |  |  |  |  |  |
